# Assessment of School Readiness in Chronic Cholestatic Liver Disease: A Pilot Study Examining Children with and without Liver Transplantation

**DOI:** 10.1155/2017/9873945

**Published:** 2017-01-17

**Authors:** Anna Gold, Alaine Rogers, Elizabeth Cruchley, Stephanie Rankin, Arpita Parmar, Binita M. Kamath, Yaron Avitzur, Vicky Lee Ng

**Affiliations:** ^1^Transplant and Regenerative Medicine Centre, Hospital for Sick Children, Toronto, ON, Canada; ^2^Department of Psychology, Hospital for Sick Children, Toronto, ON, Canada; ^3^Department of Rehabilitation, Hospital for Sick Children, Toronto, ON, Canada; ^4^Department of Occupational Science and Occupational Therapy, University of Toronto, Toronto, ON, Canada; ^5^Division of Pediatric Gastroenterology, Hepatology and Nutrition, Hospital for Sick Children, Toronto, ON, Canada; ^6^Department of Pediatrics, University of Toronto, Toronto, ON, Canada

## Abstract

*Background.* Assessment of school readiness evaluates physical, social-emotional, and neuropsychological domains essential for educational success. Cognitive testing of preschool aged children with chronic liver disease may guide more timely interventions and focused efforts by health care providers.* Patients and Methods.* Children with chronic cholestatic liver disease diagnosed as an infant and still with their native liver (NL) and children who received a liver transplant (LT) before age of 2 years underwent testing with a battery of well-validated pediatric psychometric measures.* Results.* Eighteen (13 LT, 5 NL) patients (median age of 4.45 and 4.05 years, resp.) were tested. Median Full-Scale IQ was 98 (range 102–116) for LT and 116 [(range 90–106), *p* = 0.35, NS] for NL subjects. LT recipients had significantly greater visual based difficulties, poorer caregiver rated daily living skills (*p* = 0.04), and higher levels of executive function based difficulties (e.g., inattention, inhibition).* Conclusion.* This pilot study highlights the risk of neuropsychological difficulties in early school age children who were under 2 years of age at time of LT. Comprehensive early school age assessment should integrate psychometric measures to identify children at greatest risk, thus allowing for proactive educational intervention.

## 1. Introduction

The early years of life are critical to a child's brain development as they form the foundation for cognitive, social, and emotional health [1] and set the stage for subsequent academic success. The American Academy of Pediatrics (AAP) promotes evaluation of school readiness, inclusive of physical (motor and health), social-emotional, and educational skill development of young children, achieved through a combination of child, school, and family/community based intervention [1]. Liver dysfunction and disease may result in challenges impacting successful transition to school in children with cholestatic liver disease and those undergoing LT, due to risk factors such as surgical intervention, anaesthesia, medications, malnutrition, hyperammonemia, severe ascites, and infection [2–5]. Those children who subsequently require liver transplantation before the age of 2 years have additive risks including recurrent and prolonged hospitalization stays, posttransplant complications, and sequelae of life-long immunosuppression during a time of rapid developmental changes [2].

The evaluation of children at the time of school entry, with necessary modifications for those at increased risk, may be essential to future academic success [1]. The objectives of this pilot study were to assess school readiness by examining neurodevelopmental (general intellect, preacademics, and visual and motor skills), emotional (e.g., anxiety, depression, anger, and sociability), and adaptive behaviour/functioning (e.g., self-care, community skills) in a cohort of preschool children with chronic cholestatic liver disease still with native liver (NL) or in receipt of a LT before age of 2 years.

## 2. Patients and Methods

### 2.1. Participants

Eligible patients were recruited from the ambulatory hepatology or liver transplant clinics at the Hospital for Sick Children, Toronto, Canada, between November 2012 and July 2013, using convenience sample methodology. Patient inclusion criteria were as follows: (1) primary condition of chronic cholestatic liver disease; (2) present age between 3.0 and 6.11 years; (3) being able to communicate comfortably in English; (4) absence of known neurological diagnosis or preexisting significant developmental delay; (5) not hospitalized within the 4 weeks prior to testing. Patient exclusion criteria included the following: (a) primary condition of fulminant liver failure, liver cancer, or metabolic liver condition; (b) in receipt of a multiorgan transplantation; (c) being listed for LT; (d) not sufficiently fluent in English to understand and complete testing; (e) weak or abnormal muscle tone or coordination that would have severely impaired their ability to perform physical tasks during testing; (e) having been hospitalized within the past four weeks prior to testing; or (f) prior neurocognitive testing for clinical indications in the preceding 12-months.

Eligible patients were stratified to either liver transplant (LT) or native liver (NL) groups. Caregivers of eligible patients were contacted by a clinical research associate to introduce the study and outline specific aims via telephone. If assent was obtained, families were approached by a research associate at their next clinic visit for rereview of study aims and then completion of the study consent form. This study was approved by the Research Ethics Board at the Hospital for Sick Children.

### 2.2. Psychometric Measures

Test measures were administered by a trained psychometrist (EC), under the supervision of the study psychologist (AG) and the study occupational therapist (AR). The test battery was administered in the same order for all participants, with rest breaks provided as needed. Caregivers waited in a designated waiting room during assessments and completed the parent-versions of the BRIEF, BASC-II, and Vineland-II. The teacher/daycare versions of the BRIEF and BASC-II questionnaires were mailed with caregiver consent after the assessment, with a request for respondents to return response by mail directly to the study psychologist.

### 2.3. Statistical Analysis

Analyses were performed using SPSS V. 9.3. Demographic characteristics of the participants were analyzed with appropriate descriptive statistics. For continuous variables, median and interquartile comparisons were used for all tests. Statistical analyses for this study compared median scores between groups due to the conservative nature of these tests that support a small sample size and minimize the confounding effect of outlying observations. Mean score of each psychometric measure was 100 with a standard deviation of 15, thus standard scores between 70 and 85 would fall within one standard deviation of the mean, and scores < 70 would fall at two-standard deviations below the mean. All tests employed normative age-matched data, with Canadian norms employed, where available. All test scores were converted into standard scores for statistical analysis consistency and are presented as median values.

A nonparametric permutation test was used to compare the differences of outcomes between LT and NL groups on psychometric measures and caregiver and teacher questionnaires. A nonparametric permutation test was similarly used to compare scores between teacher and caregiver groups for the BRIEF and BASC-II. A Monte Carlo simulation was used to estimate exact *p* value between these groups. To compare caregiver and teacher scores separately within LT and NL groups, a nonparametric Sign Rank test was used to compare score outcomes. All comparisons were analyzed with a statistical significance of <0.05.

## 3. Results

### 3.1. Participants

Twenty-seven patients were approached in the hepatology and LT clinics. One LT patient declined participation, five NL patients were unable to accommodate testing sessions, and one NL patient had received prior neuropsychological assessment just prior to the commencement of this pilot study, leading to a total of 20 (15 LT, 5 NL) participants tested. Two LT patients were unable to complete testing due to significant behavioural issues, leaving a study cohort of 18 (13 LT) patients available for data analysis. Of those not included, the 3 transplant patients were diagnosed with Biliary Atresia, with a mean total length of hospital stay of 111 days (range 85–152 days), and, of those 6 patients with native liver disease, 3 were diagnosed with Biliary Atresia and 3 with progressive familial intrahepatic cholestasis (mean total length of hospital stay of 27 days, with range of 6–46 days). At this institution during the study time frame, there were 2 LD patients and 10 LT patients that would have been eligible but were not approached. Within the final study cohort, the median age at testing was not statistically different between the LT [4.69 (range 3.03–6.97) years] and NL subjects [4.49 (3.47–6.26) years].  Table 1 summarizes the patient demographics and medical characteristics. Within the transplanted group, none of the patients had retransplantation, although 9 (62%) had experienced an episode of rejection by the time of testing. At the time of testing, patients in the NL group had been hospitalized for a mean total of 16.75 days (range 5–25), and none had ascites diagnosed on ultrasound. No patients in either group had clinical evidence of hepatic encephalopathy.

### 3.2. Neurocognitive Outcomes

#### 3.2.1. WPPSI-IV

Full-Scale IQ (FSIQ) scores were not significantly different between the LT [98 (102–116)] and NL [116 (90–106), *p* = 0.35, NS] participants, who were at or above age expected levels (Table 2). LT subjects had lower median scores on tasks of visual constructional [Visual-Spatial Index (VSI): 90] and visual reasoning abilities [Fluid-Reasoning Index (FRI): 97] compared to the NL group participants (VSI: 106, FRI: 112). However, LT participants demonstrated a greater scatter of scores across both the VSI and FRI (IQR: 24 and 27 standard scores points, resp.) compared to the NL group (15 and 18 points, resp.). Similar age expected scores were obtained for both groups for tasks of language, processing speed, and working memory (*p* = 0.36, *p* = 0.15, and *p* = 0.79, resp.).

#### 3.2.2. Bracken and NEPSY-II

Understanding of preacademic concepts was within the “average-high average” range for both LT (standard score: 109) and NL (standard score: 113) participants. Only 4 of 13 (30%) LT and 3 of 5 (60%) NL participants completed NEPSY-II testing, and thus statistical analysis was not possible with this number of missing variables.

#### 3.2.3. Beery-Buktenica Test

**Figure 1:**
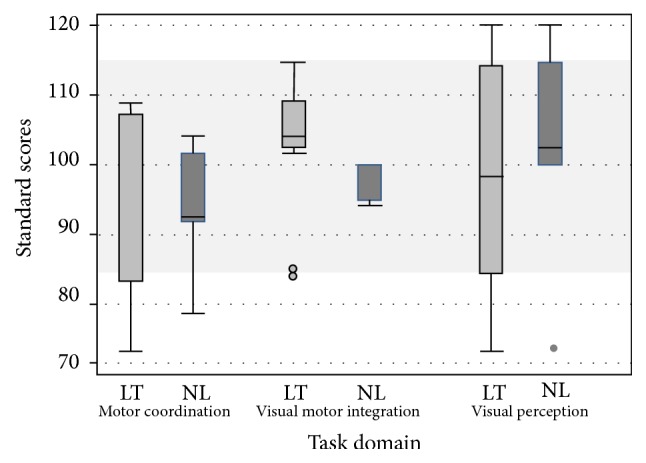
Beery VMI score results between LT and NL groups.

Median scores for LT and NL study participants were similar for tasks assessing drawing (104, 100), tracing (100, 93), and visual reasoning abilities (97.5, 103). On the visual perception subtest, 6 out of 13 (46%) LT subjects had scores below 90 (“low average” range), with only one participant in the NL group falling within this range. There was a trend towards lower scores for the LT group on all three subtests (between-groups difference of 4–7 standard score points). The IQR for the visual perception and motor coordination subtests was considerably greater for the LT group (29.5 and 23, resp.), when compared to the NL group (14 and 10, resp.), suggesting increase within group discrepancy among the LT cohort (Figure 1).

#### 3.2.4. Movement Assessment Battery for Children

**Figure 2:**
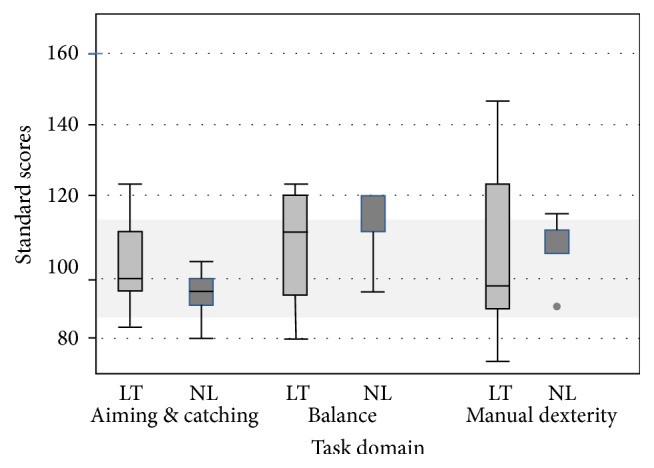
Movement ABC score results between LT and NL groups.

NL participants obtained a standard score of 120 on the balance component, greater than one standard deviation above age expected norms, in contrast to the LT group obtaining lower albeit age expected scores (standard score: 107.5, *p* = 0.40 NS). LT participants had considerably greater dispersion on the balance and manual dexterity subtests compared to the NL group (difference of 13 and 31.5 standard score points, resp.), suggesting greater variability in performance within the LT group (Figure 2).

### 3.3. Questionnaire Results

#### 3.3.1. Vineland-11

Table 3 provides parental reported scores for each of the LT and NL participant groups, with no statistically significant findings on the test domains of adaptive behaviour, daily living,and sociability skills. Parents of LT participants reported lower scores for communication (e.g., listening, understanding, talking, reading, and writing), motor skills, social skills, and self-care. NL participants displayed very strong communication skills (standard score: 129.5), with LT participants scoring in normal range for age (standard score 106, *p* = 0.0078). Fine and gross motor skills and sociability abilities (e.g., coping skills, interpersonal relationships) were also rated as above average in the NL group with a median score of 112.5 and 117, respectively), when compared to LT participants who fell in the average range [median 100 and 105, resp., (*p* = 0.03; 0.08, NS)].

#### 3.3.2. BASC-II and BRIEF

**Figure 3:**
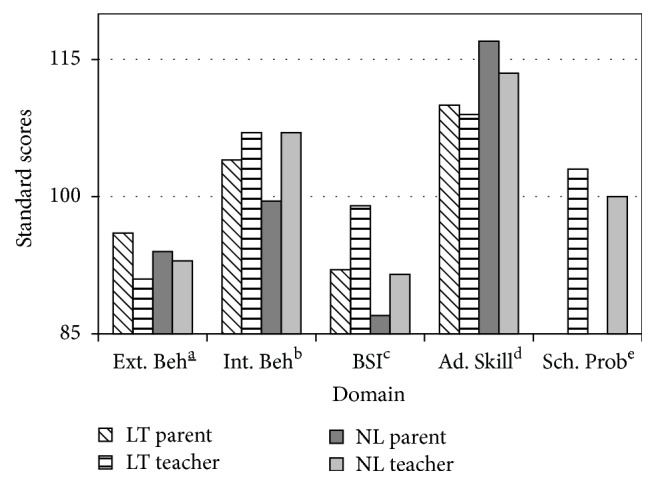
BASC-II parent and teacher scores between LT and NL groups. Higher standard scores represent greater perceived skill deficit, with the exception of adaptive skills, where lower scores represent poorer perceived level of functioning. Externalizinga_ behaviour. ^b^Internalizing behaviour. ^c^Behaviour Symptoms Index. ^d^Adaptive skill. ^e^School problems.

**Figure 4:**
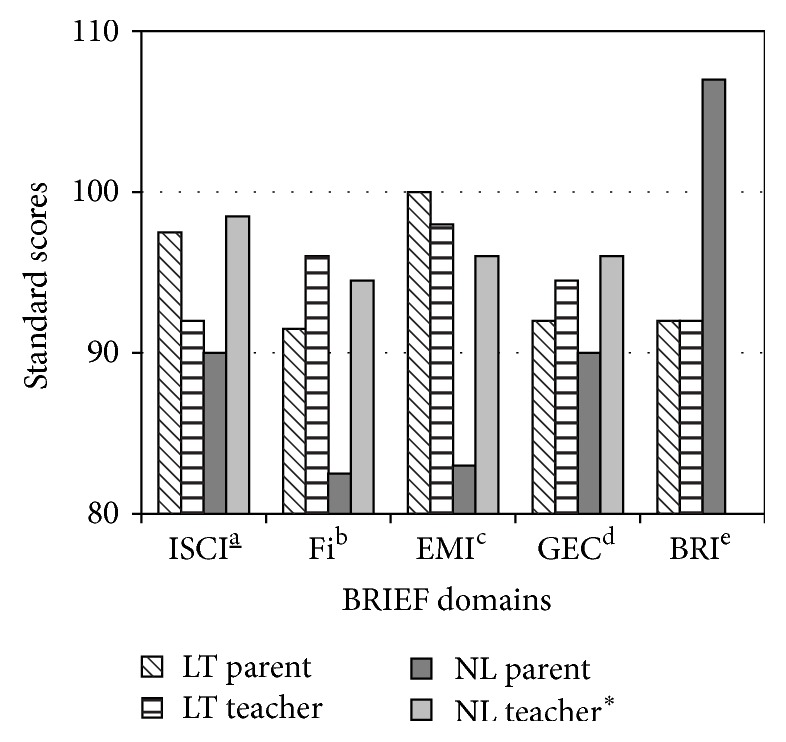
BRIEF parent and teacher scores between LT and NL group. ^*∗*^Higher scores represent poorer perceived functioning. Inhibitorya_ Self-Control Index. ^b^Flexibility Index. ^c^Emergent Metacognition Index. ^d^Global executive composite. ^e^Behavioural Regulation Index.

Parental reports of externalizing, internalizing, and adaptive behavioural domains from the BASC-II were not statistically different* across groups *(Figure 3). However, teachers endorsed an overall greater prevalence of internalizing problems (e.g., anxiety, low mood, and somatization) when compared to parents, most notable among the NL group (*p* = 0.05). On the BRIEF questionnaire (Figure 4), parents of LT children reported higher levels of inattention (standard score 100), including inhibitory self-control (standard score 97.5) and cognitive flexibility (standard score 91.5), compared to their NL counterparts (standard scores 83, 90, and 82.5, resp., *p* = 0.04). As seen on the BASC-II questionnaire, teacher ratings on the BRIEF were generally higher compared to parents, suggesting greater perceived difficulties across most domains for both groups.

## 4. Discussion

This pilot study evaluating school readiness in a targeted cohort of patients with chronic cholestatic liver disease demonstrated that those who underwent LT have a higher prevalence of visual and motor based difficulties and have challenges affecting motor skills, communication, and executive functioning skills as reported by their parents. In addition, teachers reported greater executive difficulties and emotional issues (e.g., anxiety, low mood) across both groups (when compared to healthy classmates).

We identified specific visuospatial and constructional difficulties in our cohort of young children with chronic cholestatic liver disease [3, 6]. Knowledge of early concepts such as shape, number, and letters fell at or above age expected norms among our cohort, using the Bracken measure which assesses* understanding *of early concepts, not emergent reading, spelling, or number abilities. Previous studies have reported a 3-fold increase in learning disabilities in pediatric liver transplant [4, 5]. This higher prevalence may be associated with the negative impact of visuospatial difficulties on early academic skill acquisition/production. These difficulties may persist through later school years, as evidenced by decreased overall intelligence quotient (IQ) and neuropsychological dysfunction [7–9] specifically for visuospatial skills [10], executive [11], behavioural, and emotional functioning [12–14] in medium-long term survivors of pediatric LT.

Visuospatial difficulties have been correlated with pretransplant growth deficits and increased serum ammonia [15]. Academic achievement was less delayed in children who had transplant at a younger age with fewer growth deficits [11]. In our group, the average intellectual abilities could be related to young age at transplant and high proportion (70%) of live donor liver transplantation, with shorter wait time and stringent study criteria, such as excluding patients with non-English speaking parents and abnormal muscle tone may have positively influenced the selection of patients. In adults, fewer reported neurological complications are observed in patients with living (versus deceased) donor organs, thought to be associated with enhanced graft health and shorter cold ischemic time [16, 17] and lower use of neurotoxic immunosuppression [18, 19]. Brain imaging documents diffuse cortical and subcortical involvement in adult patients with cirrhosis secondary to viral etiologies, associated with reduced blood flow and metabolic insufficiencies [16], and specific hypoperfusion in the caudate, thalamus, and cerebellum [20]. Pediatric neuroimaging literature in this field is scarce and not routinely incorporated into research protocols but nevertheless extremely important as the developing brain is much more vulnerable to injury.

In our study, parents of LT recipients report greater difficulties with emergent metacognitive skills compared to their counterparts with no transplantation, highlighting evolving working memory, task initiation, and planning/organization challenges. Teachers report increased behavioural dysregulation and inflexibility in both groups when compared to healthy classmates. In several larger population studies [12, 21] evidence of sustained attention, working memory, and behavioural regulation deficits has been reported, less often at home compared to school, following pediatric LT. Greater than 30% of 13-year-old children with transplantation [4] were diagnosed with Attention Deficit Hyperactivity Disorder (ADHD), which is much higher than general reported prevalence of 5%. Interestingly, none of the siblings in this cohort shared this diagnosis, suggesting a possible acquired (not hereditary) etiology. School may place greater demands on executive skills (e.g., adhering to classroom structure/routine, social interaction, and sustained attention to teacher instruction) compared to home. Executive abilities are not fully developed until late teens/early 20s; behavioural dysregulation is common in younger children, with cognitive difficulties emerging with increasing age (e.g., working memory, organization, planning, and task completion) [16]—reflecting the neurological maturation of these skills. The observed behavioural dysregulation during testing and from teacher report in our study may reflect the younger age of our sample and underscores the need for ongoing screening of broader (cognitive) executive difficulties.

Despite normal overall motor competence, manual dexterity, and ball skills, LT subjects scored lower on balance skills. Poorer balance skills following transplant may relate to the impact of end stage liver disease (significant abdominal distension) and transplant surgery (affecting core musculature and higher-level balance skills). A study [20] utilising subjective parent report of motor functioning in LT indicated age appropriate skills, but results were not corroborated with formal motor testing. Almaas et al. [17] assessed 35 liver transplant recipients (4–12 years, median 5.1 years after transplant) and found significantly poorer manual dexterity and ball and balance skills compared to healthy controls. Follow-up testing 4 years later indicated persistent motor weakness with a decline in ball skills. In our cohort, the LT group demonstrated strong motor skills, which could be attributed to their young age where task demands are lower, as well as the fact that almost two-thirds (61%) of our cohort were referred to community services upon discharge. Earliest intervention (occupational therapy and physiotherapy) should be considered.

In our study, teachers endorsed higher levels of internalizing issues (e.g., low mood, somatization) in both groups compared to parents. Potential contributory factors include the following: higher levels of school stress due to the need to navigate academic and social demands and managing a chronic medical condition [18]. The negative impact of chronic illness on the whole family unit [4] is reported in traumatic brain injury [15]. Self-report and parent report of health related quality of life in LT and renal transplant [18] indicate lower overall scores when compared to healthy children [19], which is similar to our study where the parents of LT children report lower academic, social, and motor competency compared to LD parents. It could be hypothesized that childhood medical illness leading to organ failure/transplant could result in a complex neurodevelopmental profile affecting the neuronal maturation of multiple brain regions. The child's early psychosocial environment such as prolonged and frequent hospitalization, frequent school absence, limited peer interactions, and family stressors also intuitively plays a contributory role in neuropsychological adaptation.

Thus, the following clinical interventions are recommended: regular school and recreational based physical activity during the pre- and posttransplant phase; the provision of information to families and schools regarding the potential impact of liver disease and transplant on neurodevelopmental functioning; and regular liaison between the family, school, and health care teams. This approach needs to sensitively balance the provision of necessary and appropriate support with potentially inappropriate limitations that could hinder opportunities for physical and cognitive development. Caregivers may not endorse educational or behavioural difficulties, which may not be obvious to the medical team during often busy clinic visits, making the routine provision of comprehensive multidisciplinary team assessment a priority.

The strengths of this study are the homogeneous patient population of a targeted young age range which allows for early identification and remediation. NL was chosen as a comparison group as it is thought to better represent the relevant medical issues compared to other clinical groups such as cystic fibrosis [5]. Study limitations include the following: a small overall sample size (associated with the narrow time frame for this pilot study); unequal numbers between the 2 groups, often seen in convenience sampling, which limits the statistical power necessary to detect differences between the groups. Attempts to mitigate this were made through rigorous statistical analysis, employing median values to overcome bias of sample size and variability across the data set. In addition, the strict inclusion/exclusion criteria may well have excluded patients with more extensive developmental and/or neurological concerns. The demographic data suggests a relatively higher level of maternal education and household income than is typically seen in our culturally diverse region.

In conclusion, liver disease and transplant can impact the acquisition of early motor, cognitive, and academic proficiency. Careful attention to school readiness and school liaison during routine clinical follow-up is essential, allowing for early identification of “at-risk” students, reducing the likelihood of prolonged academic struggles and associated emotional/behavioural sequelae. Access to early rehabilitation support (e.g., PT, OT, psychology, speech, and language) will allow for regular surveillance before and after transplant; this type of “wrap-around care” is important for children considered to be at greatest developmental risk, such as those with direct neurological involvement, and/or greater metabolic instability. Future larger, multicenter, or longitudinal studies are needed to enhance generalizability towards the overarching goal of improving positive outcomes for young pediatric transplant patients.

## Figures and Tables

**Table 1:** Demographic data.

	LT *n* = 13	NL *n* = 5
	*N* (%)	*N* (%)
*Gender *		
Female	8 (61)	4 (80)
*Age at testing*	4.45 (3.03–6.97)	4.07 (3.47–6.26)
*Median, range [years]*		
*Race *		
White	6 (46)	2 (40)
Black	1 (7)	
Asian	3 (23)	3 (60)
Others/not known	3 (23)	
*Diagnosis*		
Biliary Atresia	13 (100)	4 (80)
Alpha 1 antitrypsin		1 (20)
*Mean body mass index at time of testing (range)*	16.7 (14.4–20.8)	16.58 (15.3–17.3)
*Attending school/daycare *	11 (85)	3 (60)
*Maternal education *		
High school graduate	5 (38)	1 (20)
Postsecondary	4 (30)	4 (80)
Postgraduate	2 (15)	0
Not available	2 (15)	
*Annual family income*		
Under $50,000	2 (15)	3 (60)
$50,001–$80,000	1 (7)	2 (40)
Over $80,001	7 (54)	0
Not available	3 (23)	
*Type of immunosuppression*		
Tacrolimus	11 (85%)	
Sirolimus	2 (15%)	
*# of rehabilitation service visits*		
1–3	8 (62%)	
4–7	2 (15%)	
>7	1 (7)	
*Age at transplant*		
<12 months	7 (54%)	
12–23 months	6 (46%)	
*Type of transplant*		
Whole	1 (8%)	
Reduced	1 (8%)	
Split	2 (15%)	
Live donor	9 (69%)	
*Mean bilirubin at transplant (micromole/L) (range)*	45.5 (0–131)	
*Mean total days in hospital, before transplant (range)*	48.7 (0–172)	
*Mean total days in hospital, after transplant (range)*	65.3 (15–223)	
*Mean total days in hospital (range)*	114 (0–223)	16.75 (5–25)
	*At time of transplant*	*At time of testing*
*Mean weight (kg) (range)*	8.17 (4.78–13.93)	16.18 (14.7–17.6)
*Mean INR (range)*	1.4 (1–1.9)	0.94 (0.9–1)
*Mean albumin levels (g/L) (range)*	29 (22–36)	44.6 (37–49)
*Medications (number of patients)*		
*URSO*		80% (4)
*Septra*		80% (4)

**Table 2:** Cognitive task results, WPPSI-IV.

Test domain	Group	*N*	Median	IOR	*p* value
Verbal Composite Index	LT	11	96	15	0.36
	NL	5	102	18	
Visual-Spatial Index	LT	12	90	24	0.16
	NL	5	106	15	
Fluid-Reasoning Index	LT	7	97	27	0.56
	NL	3	112	18	
working Memory Index	LT	11	106	17	0.79
	NL	5	106	22	
Processing Speed Index	LT	7	100	17	0.15
	NL	3	108	25	
Full-Scale IQ	LT	11	98	17	0.35
	NL	5	116	25	

**Table 3:** Vineland scores for LT and NL groups.

Test domain	Group	*N*	Median	*p* value
Adaptive behavioural composite	LT	10	105	0.10
	NL	4	119	
Communication	LT	10	106	0.01^*∗*^
	NL	4	129.5	
Daily living	LT	11	109	0.83
	NL	4	113	
Sociability	LT	11	105	0.08
	NL	4	117	
Motor skills	LT	10	100	0.02^*∗*^
	NL	4	112.5	

^*∗*^Statistically significant scores (<0.05).

## Appendix

See Tables 1–3 and Figures 1–4.
