# Corrigendum to “Assessment of School Readiness in Chronic Cholestatic Liver Disease: A Pilot Study Examining Children with and without Liver Transplantation”

**DOI:** 10.1155/2017/3125082

**Published:** 2017-07-12

**Authors:** Anna Gold, Alaine Rogers, Elizabeth Cruchley, Stephanie Rankin, Arpita Parmar, Binita M. Kamath, Yaron Avitzur, Vicky Lee Ng

**Affiliations:** ^1^Transplant and Regenerative Medicine Centre, Hospital for Sick Children, Toronto, ON, Canada; ^2^Department of Psychology, Hospital for Sick Children, Toronto, ON, Canada; ^3^Department of Rehabilitation, Hospital for Sick Children, Toronto, ON, Canada; ^4^Department of Occupational Science and Occupational Therapy, University of Toronto, Toronto, ON, Canada; ^5^Division of Pediatric Gastroenterology, Hepatology and Nutrition, Hospital for Sick Children, Toronto, ON, Canada; ^6^Department of Pediatrics, University of Toronto, Toronto, ON, Canada

In the article titled “Assessment of School Readiness in Chronic Cholestatic Liver Disease: A Pilot Study Examining Children with and without Liver Transplantation” [1] there was an error in the Abstract, where the statement “Median Full-Scale IQ was 98 (range 102–116) for LT and 116 [(range 90–106), *p* = 0.35, NS] for NL subjects” should be corrected to “Median Full-Scale IQ was 98 (range 90–106) for LT and 116 [(range 102–116), *p* = 0.35, NS] for NL subjects.”
